# Clostridioides difficile Infection in Patients with Chronic Kidney Disease: A Systematic Review

**DOI:** 10.1155/2021/5466656

**Published:** 2021-09-13

**Authors:** Adelina Mihaescu, Arlyn Maria Augustine, Hassan Tahir Khokhar, Mohammed Zafran, Syed Shah Mohammed Emmad Masood, Georgiana-Emmanuela Gilca-Blanariu, Adrian Covic, Ionut Nistor

**Affiliations:** ^1^Department of Internal Medicine II-Division of Nephrology, “Victor Babeș” University of Medicine and Pharmacy Timisoara, Romania; ^2^Centre for Molecular Research in Nephrology and Vascular Disease, Faculty of Medicine, “Victor Babeș” University of Medicine and Pharmacy, Romania; ^3^University of Medicine and Pharmacy “Grigore T. Popa”, Iași, Romania; ^4^Gastroenterology Department, University of Medicine and Pharmacy “Grigore T. Popa”, Iași, Romania; ^5^Nephrology Department, Dr. C.I. Parhon Hospital, Iasi, Romania; ^6^Methodological Center for Medical Research and Evidence-Based Medicine, University of Medicine and Pharmacy “Gr. T. Popa”, Iași, Romania

## Abstract

*Clostridioides difficile* infection (CDI) is a health issue of utmost significance in Europe and North America, due to its high prevalence, morbidity, and mortality rate. The clinical spectrum of CDI is broad, ranging from asymptomatic to deadly fulminant colitis. When associated with chronic kidney disease (CKD), CDI is more prevalent and more severe than in the general population, due to specific risk factors such as impaired immune system, intestinal dysmotility, high antibiotic use leading to disturbed microbiota, frequent hospitalization, and PPI use. We performed a systematic review on the issue of prevention and treatment of CDI in the CKD population, analysing the suitable randomized controlled cohort studies published between 2000 and 2021. The results show that the most important aspect of prevention is isolation and disinfection with chlorine-based solution and hydrogen peroxide vapour to stop the spread of bacteria. In terms of prevention, using Lactobacillus plantarum (LP299v) proved to be more efficient than disinfection measures in transplant patients, leading to higher cure rates and less recurrent episodes of CDI. Treatment with oral fidaxomycin is more effective than with oral vancomycin for the initial episode of CDI in CKD patients. Faecal microbiota transplantation (FMT) is more effective than vancomycin in recurrent CDI in CKD patients. More large-sample RCTs are necessary to conclude on the best treatment and prevention strategy of CDI in CKD patients.

## 1. Introduction

*Clostridioides difficile* (CD), a Gram-positive spore-forming bacterium, is the leading cause of healthcare-associated diarrhea [1]. CD infection (CDI) is a health issue of immense significance due to its developing prevalence and rising mortality and morbidity [2]. Over the past decade, CDI incidence has increased dramatically in Europe and North America, with a significant increase of age-adjusted mortality rate (from 2.0 to 2.3 deaths per 100 000 population in USA) [3, 4].

The clinical presentation of CDI can range from asymptomatic to fulminant pseudomembranous colitis, septic shock, and death [5]. The pathogenesis and severity of CDI rely on the ability of CD to sporulate and germinate, the damage produced by the interaction between CD toxins (A and B) and the intestinal epithelium, the intestinal microbiota, and the inflammatory immune response of the host [6].

So far, the known risk factors for CDI include antibiotic use [7], older age [8], gastric acid suppression therapy [9], immunosuppression [10], and prolonged hospitalization [11].

Chronic kidney disease (CKD) is a worldwide epidemiology burden, estimated at an alarming 8–16% prevalence [3].

A recent meta-analysis concluded that CKD and end-stage renal disease (ESRD) patients have a 1.95-fold and a 2.63-fold higher risk of CDI than the general population, respectively [12]. In addition, CDI–related mortality is 2.15-fold higher in CKD patients [13].

The main culprit for the frequency and severity of CDI among CKD patients seems to be the immune system impairment of these patients with an inadequate response to C. difficile toxins [12]. Other risk factors for CDI present in CKD patients are intestinal dysmotility, increased use of antibiotics leading to disturbed microbiota, frequent hospitalization, immunosuppression therapy, and high PPI use [14–16].

According to recent guidelines from the Infectious Diseases Society of America (IDSA) and the Society for Healthcare Epidemiology of America (SHEA), the recommended treatment of an initial CDI episode to ensure the complete resolution of the symptoms is either vancomycin or fidaxomycin over metronidazole [17]. Although the treatments are efficacious, metronidazole and fidaxomycin are associated with recurrence rates of 20–25% [18–20]. Compared with metronidazole and vancomycin, fidaxomycin has minimal effects on the normal intestinal microbiota, thereby reducing the risk of C. difficile colonization, reinfection, and recurrence [21–23].

Since the intestinal microbiota is of utmost importance in the resolution and prevention of CDI, faecal microbiota transplantation (FMT) is an emerging therapy, recommended especially for patients with recurrent and even refractory CDIs. The procedure consists of transplanting faecal bacteria from a healthy donor in order to restore their normal colonic flora by replacing the pathogenic organisms with harmless bacteria [24]. FMT is on the spot because of its remarkable efficacy in treating recurrent CDI with a resolution ranging from 81-94% [25, 26].

Considering the epidemiological importance of C. difficile infection in CKD patients, the aim of this review is to explore the possibilities of prevention and treatment of CDI in patients with chronic kidney disease. To fulfil our aim, we conducted a meta-analysis on clinical studies analysing CDI treatment and prevention issues in CKD patients.

## 2. Materials and Methods

### 2.1. Data Source and Search Strategy

We used MEDLINE, PUBMED, and SCOPUS databases to search for English language articles that reported up to April 2021.

### 2.2. Keywords for the Search

During our search, we used keywords for the population of interest and intervention of interest which are shown in Table 1.

Articles that were considered suitable by title and a thorough abstract reading were included for full-text evaluation. Referenced articles in the selected studies were also read thoroughly for any significance.

### 2.3. Inclusion Criteria

Initially, we included all randomized controlled trials and cohort studies that reported an intervention to prevent or to treat Clostridium difficile infection in CKD patients. We searched for prophylactic antibiotherapy, triple therapy, and faecal microbial transplant.

### 2.4. Exclusion Criteria

We established a timeline from 2000 to 2021, so any article prior to this date was excluded. We considered that any manuscript older than 20 years discussed a practice that is outdated and does not have any significance in current practice. The exposure to antibiotics and risk factors for this infection has changed in this period.

### 2.5. Outcome of Interest

The primary outcome of interest of this systematic review was the prevention or treatment of prevalent CDI in patients with chronic kidney disease. The assessment of treatment was based upon the time to resolution of diarrhea (TTROD) and the response at end of therapy (EOT) in the treated groups. Additionally, the efficacy of the treatments was compared to each other by looking at the recurrence rates in various groups depending on the severity of their CKD.

### 2.6. Quality Assessment

The study quality of our randomized controlled trial was done based on Cochrane Risk of Bias tool [27], by selection bias (method of recruitment, proper method of randomization at baseline, concealment of treatment allocation, similarity of groups at baseline, and provision of eligibility criteria), detection bias (use of masked outcome assessment, blinded administrator, and blinded patients), attrition bias (incomplete data assessment, selective outcome reporting, use of intention-to-treat analysis), and other (sponsor bias).

The Newcastle-Ottawa Scale [28] was used to assess the quality of nonrandomized studies. We estimated the quality of the selection of the study groups, the comparability of the groups, and the ascertainment of the outcome.

### 2.7. Data Extraction

Data extraction and analysis including all relevant information from the text, figures, tables, and charts were evaluated by the reviewers, and studies that presented duplicate information were included only once. The extracted data included baseline characteristics, population, and the study period. The total number of CKD patients with CDI was extracted. The methods of intervention, duration of diarrhea, severity of infection, recurrence rate, time until recurrence, and outcome measures were also included. The recurrence CDI episodes were defined as the return of >3 unformed bowel movements per 24 hours and a positive stool toxin test.

## 3. Results and Discussion

### 3.1. Study Characteristics

**Figure 1:**
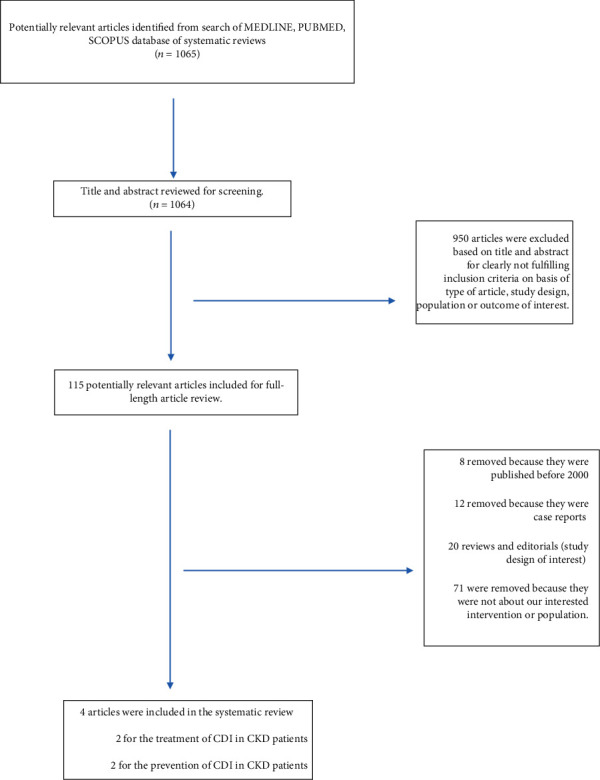
Results flow chart.

Our initial search generated 1064 articles and after title and abstract review, 950 articles were excluded leaving 114 articles for full-length review—Figure 1.

After thorough evaluation and full-text screening, only four studies were included in our analysis for prevention and treatment.  Table 2 gives an outline of these studies in terms of the author, year of publication, origin of study, study design, population, study period, total number of patients, number of patients with CKD, intervention, and the outcome.

### 3.2. Types of Interventions

Out of the 4 included studies, two studies were randomized controlled trials that focused primarily on the treatment of CDI associated with CKD, while two studies were nonrandomized controlled trials focusing on prevention.

### 3.3. Outcome Measures

Three studies (two RCTs and one of the observational non RCT) focused mainly on mortality and recurrence. One of the non-RCT focused on the severity of recurrent CDI by monitoring the duration of diarrhea, number of stools per day, and average CRP. Generally, the recurrence of CDI was mentioned in the majority of studies as a parameter to monitor the efficacy of the treatment or prevention they used. Recurrent CDI was defined as the onset of infection 28 days after the symptoms of the previous episode were completely resolved. Outcome measures are summarized in Table 3.

### 3.4. Prevention

The two studies included under the heading of prevention were by Kujawa et al. and Lachowicz et al. [29, 30] reported a cure rate of 44.4% whereas Kujawa et al. reported a higher rate of 90.4%. In addition to this, Lachowicz et al. also reported a high recurrence rate of 75% in comparison to a very low 9% reported by Kujawa et al. also included the clinical features which emphasised the severity of recurrent CDI in the 2 patients. This was measured by duration of diarrhea (days) which was decreased by 18.5 days (33.9%) and the average CRP serum concentration which showed a significant decrease from 96.5 mg/l to 43.8 mg/l.

### 3.5. Treatment

The two RCTs included under the heading of treatment compared the use of fidaxomycin, vancomycin, and faecal microbiota transplantation in stages 3-4 CKD.

In the RCT carried out by Mullane et al., the cure rate in stage 3 CKD was 80.5% with vancomycin and 79.5% with fidaxomycin. In stage 4, similar results were reported with a cure rate of 76% with vancomycin compared to 73.9% with fidaxomycin.

However, the treatment with faecal microbiota transplantation (FMT) cure rate exceeded the antibiotics. Cammarota et al. reported a significant 90% cure rate with faecal microbiota transplantation in comparison to 63.2% with vancomycin. In addition to the cure rate, FMT proved to be the better option with a low recurrence rate of 11.1% compared to the high recurrence rate of 70.5% with vancomycin. Furthermore, the use of FMT also resulted in a low mortality rate of 10% in comparison to 31.5% with the use of vancomycin as treatment.

## 4. Quality Assessment

Quality scores of the included studies ranged from 4 to 6, with a mean quality score of 5. This corresponds to a medium-to-high quality of the included studies. The detailed scores are provided in Tables 4 and 5.

## 5. Discussion

To the best of our knowledge, this is the first systematic review that discusses the role of treatment and prevention of CDI in a CKD population. Another recent meta-analysis and review [12] gathered data on the risk of incidence and recurrence of CDI in CKD patients, but did not analyse the treatment and prevention options.

Using a nationwide hospital-based database (NHDS), Keddis et al. reported a significant hospital-associated morbidity and mortality of CKD patients with associated CDI [31]. Additionally, CDI incidence was nearly four times higher among patients with both acute and chronic renal disease in a nephrology ward at a tertiary hospital, compared to other inpatients [32]. Therefore, prophylactic and therapeutic measures are necessary in this population.

### 5.1. Treatment Issue

General guidelines for treatment in adults with CDI recommend treatment with vancomycin 125 mg 4 times daily for 10 days or fidaxomycin 200 mg twice daily for 10 days for the initial nonsevere episodes. Oral metronidazole, 500 mg three times daily, is recommended only if vancomycin or fidaxomycin are not available, while intravenous metronidazole is recommended in fulminant CDI with ileus [17].

The Mullane study, an RCT, compared the treatment recommended by the general adult population guidelines—oral fidaxomycin vs. oral vancomycin—in a CKD population. The cure ratio and sustained response rate to vancomycin and fidaxomycin declined in the later stages of CKD (stages 3 and 4), which can hint on the possible role of normal kidney function in the efficacy of this treatment. However, both drugs are poorly absorbed and remain in the faeces; hence, the bioavailability should not be affected by the abnormal kidney function. Therefore, there must be another reason for the reduced treatment efficacy in the advanced stages of CKD, one hypothesis may be the proven gut microbiota dysbiosis in CKD patients [33–35], as well as the proven altered mucosal immune response in CKD patients [36]. In this study, one of the main diagnostic criteria for the efficacy of the treatment was TTROD (time to resolution of diarrhea) and an increasing trend in TTROD was observed as the kidney function deteriorated.

Overall, fidaxomycin proved to be a more efficient treatment in CKD patients because it demonstrated increased odds of sustained response by 1.89-fold relative to vancomycin, with a significant *p* value (<0.001).

### 5.2. Prevention Issue

Regarding prevention, treatment with fidaxomycin demonstrated 54% lower odds of recurrence relative to vancomycin.

The role of fidaxomycin was also supported by Rubio Teres et al. in their study that reported a low (3.8%) recurrence of CDI in comparison to a 5% recurrence risk, with vancomycin.

Looking at the faecal microbiota transplantation (FMT), Cammarota et al. conducted a randomized clinical trial in 39 patients to compare FMT and vancomycin for the treatment of recurrent CDI. The study population consisted of 20 CKD patients, stages 3 and 4, treated with FMT. 18 of them presented a complete resolution of CDI, while recurrence was reported in 2 patients (10%). Furthermore, 13 of the 18 subjects were cured after the first infusion of FMT.

According to the guidelines, FMT is recommended in the treatment of second or subsequent recurrence of CDI and can also be considered for refractory CDI. Taking into account the promising results of Cammarota et al. and the gut microbiota dysbiosis present in CKD patients, we do think that with more studies and larger clinical trials, FMT may become a step forward in efficient treatment for CDI in CKD patients.

Lachowicz et al. highlighted the importance of CDI patients' isolation by providing a segregated area for them to separate toilets and shower facilities until discharge, regardless of their symptoms.

Furthermore, another preventive measure was mentioned based upon previous studies [37, 38] that underlined the importance of disinfection by hydrogen peroxide, which has been shown to inactivate C. difficile spores within 15 minutes.

Moreover, Fawley et al. compared the effect of different disinfectants on epidemic and nonepidemic *Clostridioides difficile* strains and found that only chlorine-based agents were successful to prevent the transmission of the bacteria [39]. This was further proven by Martinez et al. (who conducted a similar study) and supported the use of such chlorine-based disinfectant agents [40]. Lachowicz et al. successfully implemented the aforementioned measures in their population of haemodialysis patients, which helped them end the outbreak. This is another proof that disinfection should be at the core of the preventive measures against any infection.

To further elaborate on the preventive measures, we found another study that explored the use of the strain 299 v of Lactobacillus plantarum (LP299v) in CKD patients with CDI.

LP299v is a gram-positive lactic acid bacterium that was isolated from the mucosa of human intestines and is also commonly found in food products [41]. An important characteristic of this strain lies in its ability to adhere to the gut wall by mannose-dependent adhesion, which seems to play a crucial role in decreasing bacterial translocation [42–45] and preventing the adhesion of different pathogens to the intestinal epithelium [46–48]. Moreover, LP299v has been shown to reduce the symptoms of irritable bowel syndrome and gastrointestinal adverse events during antibiotics exposure [49–51].

Kujawa et al. are the first ones to report the use of LP299v in transplanted patients. The study was divided into two twelve-month intervals, before and after the initiation of LP299v agent as a prophylactic agent. The results show a significant decrease in the incidence of recurrence of CDI after the initiation of prophylaxis (*p* = 0.0001).

According to the data we extracted in Table 4, Kujawa et al. and Cammarota et al. are the only two studies that demonstrated a recurrence rate below 11%. Comparing the two studies under the heading of prevention, Kujawa et al. reported a much higher cure rate and a lower recurrence rate by using LP299v compared to Lachowicz et al. disinfection measures.

Looking at other patients with a similar immunological profile to CKD patients, we analysed the issue of C. difficile infection in cancer patients. Cancer patients also have a 2-fold higher risk of CDI, due to risk factors such as frequent hospitalization, increased use of antibiotics, immune deficiency, and disturbed microbiota [52].

Even for cancer patients, studies show a higher cure rate and fewer recurrences with fidaxomycin compared to vancomycin [53]. FMT can be safely used in the recurrence of CDI in cancer patients, with an effective rate of 86%, without serious infectious side effects [54]. However, the use of probiotics has not proven a benefit in CDI prevention, even more, and there is a potential risk of blood stream infection with probiotic microorganisms in immunocompromised patients, especially those with hematological neoplasia [55].

Considered for many years as a hospital-acquired infection, CDI epidemiology research revealed interesting data: the proportion of community-associated CDI (CA-CDI) is higher than expected, with 20 to 27% of the total CDI cases being community-associated, both in Europe and in North America [3]. CA-CDI patients have different characteristics than those that acquire CDI in the hospital: they are younger, less ill, have fewer comorbidities, use less antibiotics and gastric acid suppressors, and ribotype 027 is the most frequently found strain [56].

Diabetic patients are a population which, in terms of impaired immune response, antibiotic use, healthcare exposure, and disturbed gut microbiota are very similar to the CKD population. A review of C. difficile infection in diabetes mellitus patients [57]. emphasizes the importance of impaired colonization resistance to C. difficile (deficit of precolonization of the gut with nontoxigenic *Clostridia* species and a changed ration of *Bacteroidetes* and *Firmicutes*) as the major cause of CDI in these patients. This gut microbiota dysbiosis explains the success of faecal microbiota transplantation in diabetic patients, similar to that of CKD patients [57].

A high rate of asymptomatic colonization with toxigenic C difficile (16%) was demonstrated in geriatric patients at admission in the geriatric ward. The risk factors for colonization were previous CDI, antibiotic use, and postsurgery status. The great majority (87.5%) of the asymptomatic carriers further developed C. difficile diarrhea, leading to the conclusion that screening for C. difficile colonization prior to admission could be a very useful tool in CDI prevention in a geriatric ward [58]. In the same time, the high rate of colonization with C. difficile in geriatric patients is also an issue of gut microbiota dysbiosis, a situation which could be addressed to successfully prevent or treat CDI in these patients.

*C. difficile* diarrhea in COVID-19 patients is an emerging health issue, since the SarsCov2 virus infection produces immunodepression of the host and COVID-19 patients usually receive empirical antibiotic treatment. COVID-19 main symptoms are respiratory; however, <10% of the patients present with gastrointestinal events (vomiting, diarrhea, or abdominal pain), which raises the question of differential diagnosis with CDI [36, 59]. A recent cohort study on COVID-19 and CDI coinfection revealed a 13.5% incidence of CDI in 52 critical ill patients in Michigan, USA, out of which, 94% received empirical antibiotic therapy. Further research and patients' follow-up will conclude if the impaired immune response of the patients to SarsCov2 and/or antibiotic use in these patients is involved in the COVID-19 and C. difficile coinfection.

## 6. Limitations

This systematic review does have some limitations that need to be pointed out. The total number of CKD patients is rather low. The trials are mainly observational, with only two RCTs looking at treatment and prevention. Treatment regimen and prevention methods are heterogenic in the analysed studies, and the number of patients enrolled in each trial is rather low.

## 7. Conclusions

Our review provides an insight on the very serious problem of C. difficile infection in CKD patients, from the prevention and treatment point of view. The most important aspect of prevention is isolation and disinfection with chlorine-based solution and hydrogen peroxide vapour in order to stop the spread of bacteria. Treatment with oral fidaxomycin is more effective than with oral vancomycin for the initial episode of CDI in CKD patients. FMT has a better cure rate and a lower recurrence rate than vancomycin when used for recurrence episodes and qualifies for a safe and efficient emerging treatment that needs to be further investigated in bigger trials. Prevention of recurrence episodes can be safely addressed with LP299v, a probiotic that lowers the rate of CDI in renal transplant patients.

More large-sample RCTs are necessary to conclude on the best treatment and prevention strategy of CDI in CKD patients.

## Figures and Tables

**Table 1:** Searched keywords.

(1) Clostridium (2) Clostridium difficile (3) Difficile (4) C. difficile (5) Diarrhea (6) Pseudomembranous colitis	OR 1-6
(7) Chronic kidney disease (8) Chronic kidney failure (9) Haemodialysis (10) Dialysis (11) Chronic renal failure	OR 7-11	
(12) Treatment (13) Antibiotics (14) Intervention (15) Prevention	OR 12-15	

**Table 2:** Study characteristics.

Author	Year	Origin	Population	Study type	Study period	Total patients	N=CKD	Intervention	Cured patients	Recurrence	Outcome measures
Mullane	2013	Department of Medicine, University of Chicago, Chicago	Renal impairment and CDI	Randomized controlled trials	2012	1054	321	Fidaxomycin 158 Vancomycin 163	252 (78.5%)	66 26%	(i) Clinical cure (ii) Sustained response (iii) Recurrence (iv) Mortality
Lachowicz	2014	Department of Medical Microbiology, Medical University of Warsaw	Dialysis patients	Retrospective non-RCT (observational study)	November 2012–December 2012	9	9	Environmental decontamination (chlorine bleach, indomethacin, octenisept, and hydrogen peroxide vapour) Isolation	4	3	(i) Spread of infection (ii) Microbiological analysis (iii) Mortality
Kujawa	2015	Department of Nephrology, Transplantation and Internal Medicine, Medical University of Silesia in Katowice	Kidney transplant and immunosuppressive therapy	Retrospective non-RCT (observational study)	October 2012–October 2013 and December 2013–December 2014	23	17	Lactobacillus Plantarum 299v	N/A	N/A	(i) C. difficile infection incidence (ii) Mortality (iii) Severity of CDI (iv) Duration of diarrhea (v) Number of stools per day (vi) Average CRP serum concentration
Cammarota [31]	2015	Department of Internal Medicine, a.Gemelli University Hospital Roma, Italy	Recurrent C. difficile patients	Randomized controlled trial	July 2013-June 2014	39	20	Faecal microbiota transplantation by colonoscopy Vancomycin	18 (90%)	2 (10%)	(i) Recurrence rate of C. difficile infection with FMT and vancomycin

**Table 3:** Outcome measures.

Outcome	Study	Participants	Results		
			Baseline	Posttesting	% change
Cure rate (no. of patients)	Mullane et al. 2013 [32]	Fidaxomycin stage 3 CKD	112	89	79.5
		Fidaxomycin stage 4 CKD	46	34	73.9
		Vancomycin stage 3 CKD	113	91	80.5
		Vancomycin stage 4 CKD	50	38	76.0
	Cammarota et al. 2015 [33]	FMT	20	18	90.0
		Vancomycin	19	12	63.2
	Lackowicz et al. 2014 [30]	Nephrology ward patients	9	4	44.4
	Kujawa et al. 2015 [29]	LP299v	21	19	90.4
					
Recurrence (no. of patients)	Mullane et al. 2013 [32]	Fidaxomycin stage 3 CKD	89	19	21.4
		Fidaxomycin stage 4 CKD	34	5	14.7
		Vancomycin stage 3 CKD	91	30	33.0
		Vancomycin stage 4 CKD	38	12	31.6
	Lackowicz et al. 2014 [30]	Nephrology ward patients	4	3	75.0
	Cammarota et al. 2015 [33]	FMT	18	2	11.1
		Vancomycin	17	12	70.5
	Kujawa et al. 2015 [29]	LP299v	21	2	9.0
					
Mortality (no. of patients)	Mullane et al. 2013 [32]	Stage 3 CKD	—	—	—
		Stage 4 CKD	—	—	—
	Lackowicz et al. 2014 [30]	Nephrology ward patients	9	2	22
	Cammarota et al. 2015 [33]	FMT	20	2	10
		Vancomycin	19	6	31.5
					
Duration of diarrhea (days)	Kujawa et al. 2015 [29]	LP299v in the patients with recurrence (*n* = 2)	28	9.5	33.9
Number of stools per day	Kujawa et al. 2015 [29]	LP299v in the patients with recurrence (*n* = 2)	8	7	—
Average CRP serum concentration (mg/l)	Kujawa et al. 2015 [29]	LP299v in the patients with recurrence (*n* = 2)	96.5	43.8	

**Table 4:** Quality assessment scores randomized trials.

Randomized controlled trials	Sequence generation	Allocation concealment	Blinding	Incomplete outcome data	Selective reporting	Intention to treat	Other
Mullane et al. (2013) [32]	—	—	—	—	—	?	NA
Cammarota et al. (2015) [33]	—	—	—	—	—	?	NA

**Table 5:** Quality assessment scores nonrandomized trials.

Nonrandomized controlled trials	Selection				Comparability		Outcome		
	Representative cohort	Selection of nonexposed cohort	Ascertainment of exposure	Not present at start	Control for most important factor	Control for additional factors	Assessment of outcome	Follow-up long enough	Adequacy of follow-up
Kujawa et al. (2015)									
	∗	∗		∗			∗	∗	∗
Lachowicz et al. (2014)									
	∗	∗		∗			∗		
